# The microbiota changes of the brown dog tick, *Rhipicephalus sanguineus* under starvation stress

**DOI:** 10.3389/fphys.2022.932130

**Published:** 2022-09-09

**Authors:** Liping An, Biswajit Bhowmick, Dejuan Liang, Penghui Suo, Chenghong Liao, Jianguo Zhao, Qian Han

**Affiliations:** ^1^ Laboratory of Tropical Veterinary Medicine and Vector Biology, School of Life Sciences, Hainan University, Haikou, Hainan, China; ^2^ One Health Institute, Hainan University, Haikou, Hainan, China

**Keywords:** Rhipicephalus sanguineus, starvation stress, coxiella, tick, microbia community, symbiotic bacteria

## Abstract

*Rhipicephalus sanguineus,* the brown dog tick, is the most widespread tick in the world and a predominant vector of multiple pathogens affecting wild and domestic animals. There is an increasing interest in understanding the role of tick microbiome in pathogen acquisition and transmission as well as in environment–vector interfaces. Several studies suggested that the tick microbial communities are under the influence of several factors including the tick species, dietary bloodmeal, and physiological stress. Compared with insects, very little of the microbial community is known to contribute to the nutrition of the host. Therefore, it is of significance to elucidate the regulation of the microbial community of *Rh. Sanguineus* under starvation stress. Starvation stress was induced in wild-type adults (1 month, 2 months, 4 months, 6 months) and the microbial composition and diversity were analyzed before and after blood feeding. After the evaluation, it was found that the microbial community composition of *Rh. sanguineus* changed significantly with starvation stress. The dominant symbiotic bacteria *Coxiella* spp. of *Rh. sanguineus* gradually decreased with the prolongation of starvation stress. We also demonstrated that the starvation tolerance of *Rh. sanguineus* was as long as 6 months. Next, Coxiella-like endosymbionts were quantitatively analyzed by fluorescence quantitative PCR. We found a pronounced tissue tropism in the Malpighian tubule and female gonad, and less in the midgut and salivary gland organs. Finally, the blood-fed nymphs were injected with ofloxacin within 24 h. The nymphs were allowed to develop into adults. It was found that the adult blood-sucking rate, adult weight after blood meal, fecundity (egg hatching rate), and feeding period of the newly hatched larvae were all affected to varying degrees, indicating that the removal of most symbiotic bacteria had an irreversible effect on it.

## 1 Introduction

Food is an essential source of nutrition for arthropods and an important external factor for survival. A long-term lack of food will affect the growth and reproduction of arthropods and may even lead to death. Symbionts in arthropods make essential contributions to nutrient supply. Such nutritional symbioses are crucial in many obligate blood-feeders, such as ticks, tsetse flies and bed bugs, where bacterial symbionts synthesize B vitamins lacking in the blood meal (Nikoh et al., 2014; Ben-Yosef et al., 2020). Bacterial cells provide related nutrients necessary for survival and reproduction, revealing an evolutionary pathway to obligate trophic symbionts in which host and symbionts merge into a coherent organism and highlighting the parasitic - mutualistic evolutionary continuum (Hosokawa et al., 2010; Lockwood et al., 2016). Similar correlational studies have been incomplete in ticks. The maternally inherited symbionts in the African soft tick *Ornithodoros moubata* provide missing B vitamins; these nutritional symbionts are critical for tick growth and survival in adulthood (Duron et al., 2018). Identifying and characterizing the tick microbiota is thus crucial to better understand tick-microbe interactions. While the microbiota of several tick species have been studied, little information is currently available on the microbiota of *Rh. sanguineus* which is the most widespread tick in the world. *Rh. sanguineus* is distributed in tropical and subtropical and temperate regions, and it breeds in human habitats (Dantas-Torres, 2010). They carry a variety of protozoa and microorganisms, including *Ehrlichiacanis, Rickettsia, Mycoplasma, Babesia microti*, etc., which cause diseases to humans and animals (Barker and Murrell, 2004), and they have the ability to transmit pathogens vertically through eggs (Blisnick et al., 2017).

Ticks molt after blood-feeding in each life cycle, and it is a very complex process. Ticks have a variety of symbionts; the common symbionts found in different ticks are those belonging to the genus *Rickettsia* in the hard tick, and the genus *Coxiella* in the *Rh. sanguineus*, and the genus *Francisella* in the soft tick (Zhang et al., 2017; Ben-Yosef et al., 2020). Compared with insects such as mosquitoes and flies, there are few studies on the endosymbionts of *Rh. sanguineus* and some studies have investigated the biological specificity of the symbionts of Ixodes tick (Jasinskas et al., 2007; Zhong et al., 2021). Each study used different antibiotics and administration methods, and determined bacterial load and phenotypic characterization in different ways. This study aimed to determine changes of gut microbiota in *Rh. sanguineus* under different levels of starvation stress. Ticks can survive in the wild for months or even years without food, which leads to the increase in tick-borne diseases (Zhong et al., 2021). Starvation stress affects the physiological response of organisms, *Coxiella* is the most common maternally inherited endosymbionts, known for its ability to provide B vitamins and cofactors to ticks, and has an impact on tick development, fecundity and metabolism (Zhong et al., 2021).We hypothesized that there would be significant changes in the gut microbiota of the tick at different starvation periods, which ultimately reduced the number of provided nutrients and hindered development. We first subjected adult ticks to starvation stress for different periods to test this hypothesis. We then investigated the effect of symbiotic elimination on developmental timing and female reproductive success after nymph feeding. Bacterial diversity in whole-body samples of adult ticks at different starvation periods was also assessed.

## 2 Materials and methods

### 2.1 Tick collection and grouping

A total of 578 ticks were collected from the stray animal shelter in Haikou City, China. All ticks were identified morphologically to species level using existing taxonomic keys, followed by molecular analysis (Latrofa et al., 2014). Unfed female and male ticks were used in this study. They were first washed once in 70% ethanol and rinsed three times in sterile water. The ticks were kept under laboratory conditions in an incubator at 27.5°C and 90% relative humidity until needed for experiments. New Zealand white rabbits were used for blood-feeding larvae and nymphs. New *Rh. sanguineus* nymphs were assigned into six groups, named unfed, unfed1, unfed3, unfed4, unfed6, and fed. Unfed group nymphs were collected for testing immediately after moulting. Groups unfed1, unfed3, unfed4. and unfed6 nymphs were collected for testing after 1, 3, 4, and 6 months starvation, respectively. Group fed nymphs were tested after blood feeding without starvation. Each group had 10–14 nymphs, and the experiment was repeated three times.

### 2.2 DNA extraction and PCR amplification

The nymphs collected from each group were washed with 75% alcohol and sterile water, and dissected to remove the mouthparts and four pairs of legs. The remaining bodies were pooled for DNA extraction for each group. The microbial DNA was extracted using the HiPure Soil DNA Kits (Magen, Guangzhou, China) according to the manufacturer’s protocols. The 16S rDNA target region of the ribosomal RNA gene was amplified by PCR (95°C for 5 min, followed by 30 cycles at 95°C for 1 min, 60°C for 1 min, and 72°C for 1 min, and a final extension at 72°C for 7 min) using primers listed in Supplementary Table S1 in the supplementary information (Guo et al., 2017). PCR reactions were performed in a triplicate 50 μL mixture containing 10 μL of 5 × Q5@ Reaction Buffer, 10 μL of 5 × Q5@ High GC Enhancer, 1.5 μL of 2.5 mM dNTPs, 1.5 μL of each primer (10 μM), 0.2 μL of Q5@ High-Fidelity DNA Polymerase, and 50 ng of template DNA.

### 2.4 Bioinformatics analysis

All purified amplicons were pooled in equimolar concentrations and paired-end sequenced on an Illumina PE250 platform. Bioinformatics analysis (OTU, community composition analysis, Indicator species analysis, α-diversity, β-diversity, and function prediction) of the raw data was performed using various software, FASTP (Chen et al., 2018) (version 0.18.0), FLSAH (Salzberg, 2011) (version 1.2.11), UPARSE (Edgar, 2013) (version 9.2.64), Krona (Ondov et al., 2011) (version 2.6), R package (Wickham and Boussiala, 2020) (version 2.2.1), QIIME (Caporaso et al., 2010) (version 1.9.1), Muscle (Edgar, 2010) (version3.8.31), FastTree (Price et al., 2010) (version2.1), Tax4Fun (Asshauer et al., 2015) (version1.0). All sequencing and bioinformatics analyses were performed using the Omicsmart online platform (http://www.omicsmart.com).

### 2.5 Tissue tropism of *Rh. sanguineus*

Relative densities of *Coxiella*-like symbionts in the tissue sample of the gonads, Malpighian tubules, salivary glands, midgut, and trunk (remaining parts) were analyzed using the SYBR green or fluorescence quantitative PCR (qPCR) approach as previously described (Lalzar et al., 2012). Briefly, qPCR was used to detect the expression levels in different tissues using fluorescence dye SYBR Green I (Roche Molecular Biochemicals, Mannheim, Germany). The reaction system consisted of 5 μL of qPCR master mix, 0.5 μL of upstream and downstream primers, 1 μL of template cDNA, and 3 μL of ddH2O. Real-time amplification detection was performed using Roche’s Lightcycler 96. The standard procedure of two-step PCR amplification was adopted for the reaction: 95°C for the 20 s, 95°C for 5 s, and 58°C for 30 s, a total of 40 cycles. Three replicates were set for each sample, and the primers used in the experiment were shown in Supplementary Table S2.

### 2.6 Antibiotic injection for ticks

To obtain *Rh. sanguineus* with reduced *Coxiella* burden, we microinjected blood fed nymphs with ofloxacin antibiotic at a dose of 30 ng/mg tick body weight and subsequently recorded the effect of nymphal and female development on health status. The selection of antibiotics was based on their previously established effectiveness against *C burnetii* (Jabarit-Aldighieri et al., 1992; Gikas et al., 1998). Another aspect is their inhibitory effect on *Coxiella* in other ticks (Ninio et al., 2015). These studies highlighted ofloxacin, rifampicin, and tetracycline as suitable candidates with substantial effects on *Coxiella* or *Coxiella*-like organisms. All drugs are broad-spectrum antibiotics that stop bacterial replication by interfering with DNA, RNA, and protein synthesis. The stock solution was diluted with sterile saline (0.9% NaCl, pH 6.5; ofloxacin and tetracycline) or 1/5 v/v dimethyl sulfoxide rifampicin), adjusted to a final concentration of 7 mg/ml. Ofloxacin was acidified and solubilized with hydrochloric acid (1:700 v/v normal saline: hydrochloric acid, pH 6.0), and all solutions were prepared and processed on a clean bench to ensure sterility. Our experiments show that different antibiotics have different potency against *Coxiella* and suggest that nymph developmental timing is affected differently by the antibiotics we tested. Subsequent experiments continued with the most potent antibiotic (ofloxacin) and estimated the effect of antibiotics on inhibiting nymph body weight, blood feeding rate, and post-feeding development (time to adult molting). To determine the effect of *Coxiella* on nymph development, new nymphs were randomly assigned to different groups with similar mean weights and variances. Within the next 36–48 h, the ticks were microinjected (Hamilton microinjector 2.5 μL) in sterile physiological saline solution with or without antibiotics and placed in the artificial culture at 27.5°C and 90% RH. In the box, the molting of nymphs was recorded every 24 h until all molted ticks became adults. Subsequent experiments were continued using an antibiotic (ofloxacin) against *Coxiella*. The effects of inhibition of *Coxiella* on nymph body weight, and post-feeding nymph development (time to adult molting) were evaluated. To determine the effect of *Coxiella* spp. on nymphal development, fully saturated nymphs were isolated from New Zealand white rabbits (collected at two subsequent 24-h intervals), weighed to the nearest 0.05 mg, and then randomly assigned to average body weight and in groups with similar variances. Over the next 24 h, nymphs were microinjected with 1.5 μL of sterile saline solution with or without antibiotics, then placed in 50 ml sterile centrifuge tubes perforated with caps and kept in the dark climate chamber (27 ± 1)°C and (85 ± 5%) relative humidity to complete the molting and development after blood feeding. During the following period, we recorded the molting of the nymphs every 24 h until all nymphs completed molting and became adults. These experiments were repeated twice.

For male-female mating and blood-feeding, molted female ticks were parasitized on New Zealand white rabbits. They were subsequently used to determine the effect of *Coxiella* on nymph blood-feeding rate, saturated blood weight, adult fecundity (hatching rate), and post-hatch larval blood-feeding rate. *Rh. sanguineus* was released to the host skin after the antibiotic treatment, the feeding period of female ticks lasted for 8–18 days.

### 2.7 Data analysis

The diversity of starvation stress time microbial communities with a supplied diet was evaluated by *16S rRNA* gene sequencing. After sequencing, we first filtered the low-quality reads from Raw reads obtained, assembled the paired-end reads into tags, and then filtered the tags. The obtained data is called Clean tag. Next, clustering is performed based on the Clean tags, the chimera tags detected during the clustering alignment were removed, and the obtained data is the Effective tag. Data preprocessing statistics and quality control were shown in Supplementary Table S3. After obtaining OTUs, the overall characteristics of each sample OTU, low abundance OTU, Tags annotation, and other information were statistically summarized based on OTU’s abundance information and species annotation information. The results were shown in Table1.

**TABLE 1:** Statistics of tags and OTUs.

Sample ID	Total tags	Taxon tags	Unclassified tags	Singleton tags	OTUs
Fed-1	110319	103558	0	6,761	227
Fed-2	111034	105161	0	5,873	170
Fed-3	104216	99075	0	5,141	167
Unfed-1	111450	105474	0	5,976	178
Unfed-2	113370	107776	0	5,594	153
Unfed-3	102057	97371	0	4,686	141
Unfed1-1	116223	107236	0	8,987	275
Unfed1-2	113451	107712	0	5,739	313
Unfed1-3	114760	107192	0	7,568	231
Unfed3-1	108206	101606	0	6,600	151
Unfed3-2	113693	106976	0	6,717	220
Unfed3-3	110401	89921	0	20480	623
Unfed4-1	108871	102902	0	5,969	221
Unfed4-2	102971	97678	0	5,293	200
Unfed4-3	102743	97416	0	5,327	152
Unfed6-1	104089	97620	0	6,469	231
Unfed6-2	104333	98382	0	5,951	215
Unfed6-3	111313	104597	0	6,716	262
Avg	109083	102091	0	6,991	229

All the data were tested for normality using the Shapiro-Wallis test. The differences between three or more groups were determined using the Kruskal-Wallis test. Other comparisons were performed using Mann Whitney test or two-tailed *t*-test depending on Gaussian distribution. The values were statistically significant when *p* < 0.05. All statistical calculations were performed using GraphPad Prism software (version 7) except the effect size, calculated using the Cohen’s d.

### 2.8 Ethics statement

The use of experimental animals was reviewed and approved by Hainan University Institutional Animal Care and Use Committee (HNUAUCC-2021-00095).

## 3 Results

### 3.1 Effects of starvation stress on microbial communities in ticks

**FIGURE 1:**
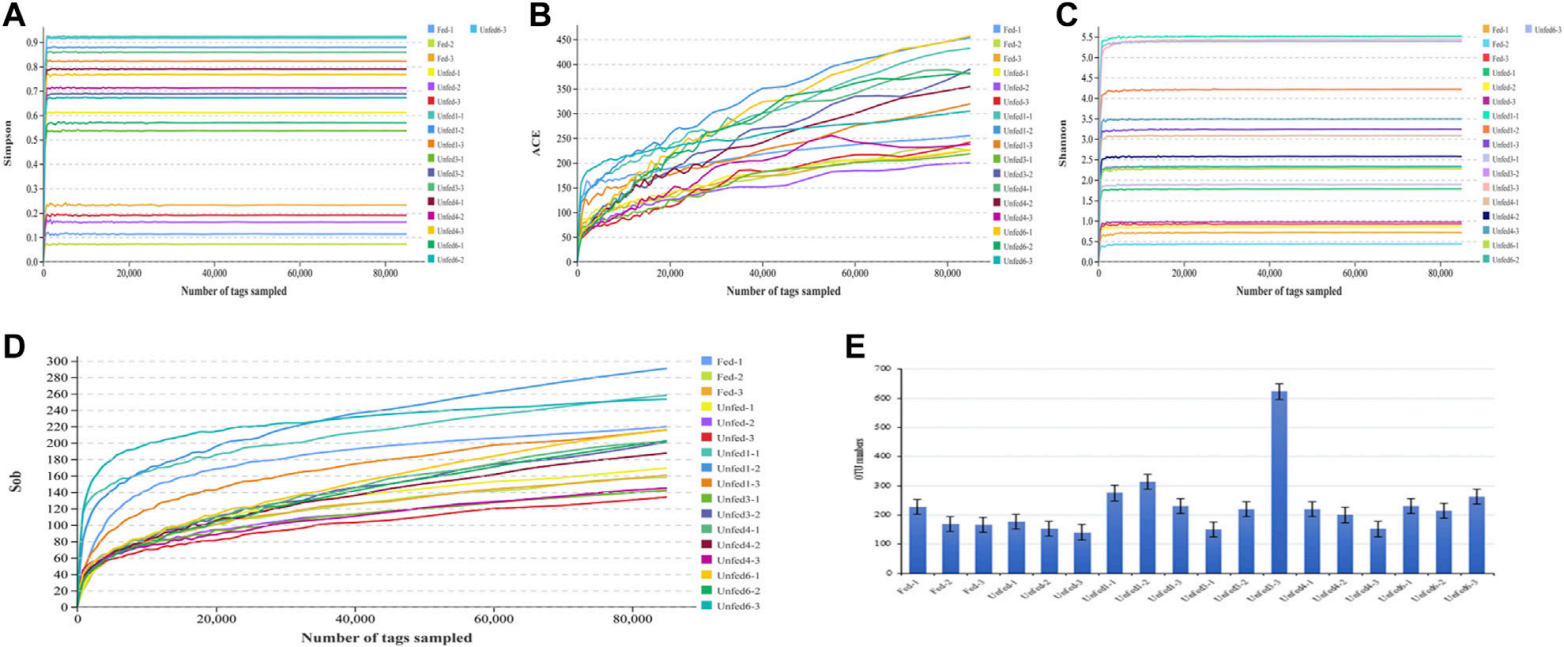
Diversity of gut microbial community as affected by starvation. Data from different groups were presented with different colors. Simpson **(A)**, ACE **(B)**, Shannon **(C)**, Sob **(D)** indices, OUT numbers **(E)** were shown in different panels. The group names were descripted in the Materials and Methods, and data from the three repeats in one group were shown separately. Note that since the correlation coefficient between the Unfed3-2 samples and the other two samples, Unfed3-1 and Unfed3-3, is low, this sample was excluded by the program before the analyses for the accuracy.

After removing the low quality or no biological significance tags, a total of 1,963,500 effective tags were acquired from the 18 samples analyzed. An average of 109,083 effective tags was covered for each sample. The effective ratio of all samples averaged 84.81% (ranging from 82.15% to 86.1%) (Supplementary Table S1). The diversity index curve that with the extension of starvation time and the curves of all samples were gradually flattened and saturated, indicating that the sequencing quality of all samples was reliable. The α-diversity indices including ACE, Shannon, Simpson, and Sob are depicted in Figure 1. The above four alpha diversity indices of the unfed group were higher than those of the fed group. Notably, species richness in the unfed group-1 was higher after the starvation treatment.

### 3.2 Microbial composition in ticks

**FIGURE 2:**
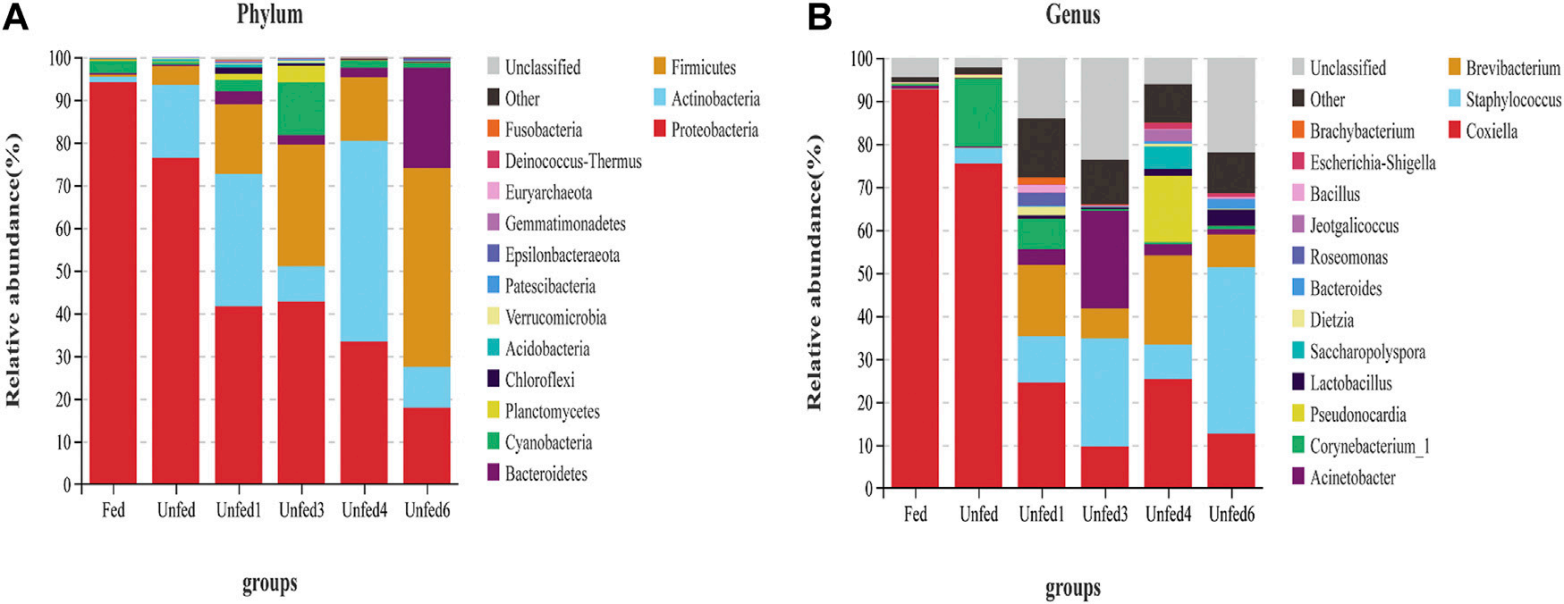
Comparison of the different microbiome Top 15 bacteria in the nymphs from each group (*n* = 3). **(A)** The microbiome at the phylum level; **(B)** The microbiome at genus level.

Different bacteria of 30 phyla, 86 classes, 129 orders, 234 families, 485 genera, and 149 species were identified in all ticks. Figure 2, shows that the total number of Proteobacteria, Actinobacteria, and Firmicutes was more than 80%. Therefore, the focus is on these three particular phyla. At the genus level, the majority of sequences belonged to *Coxiella*, followed by *Staphylococcus*, *Brevibacterium*, *Acinetobacter*, *Corynebacterium*, *Pseudonocardia*, *Lactobacillus*, *Saccharopolyspora*, and other unclassified bacteria.

### 3.3 Prediction of functional profiles of tick microbiota

**FIGURE 3:**
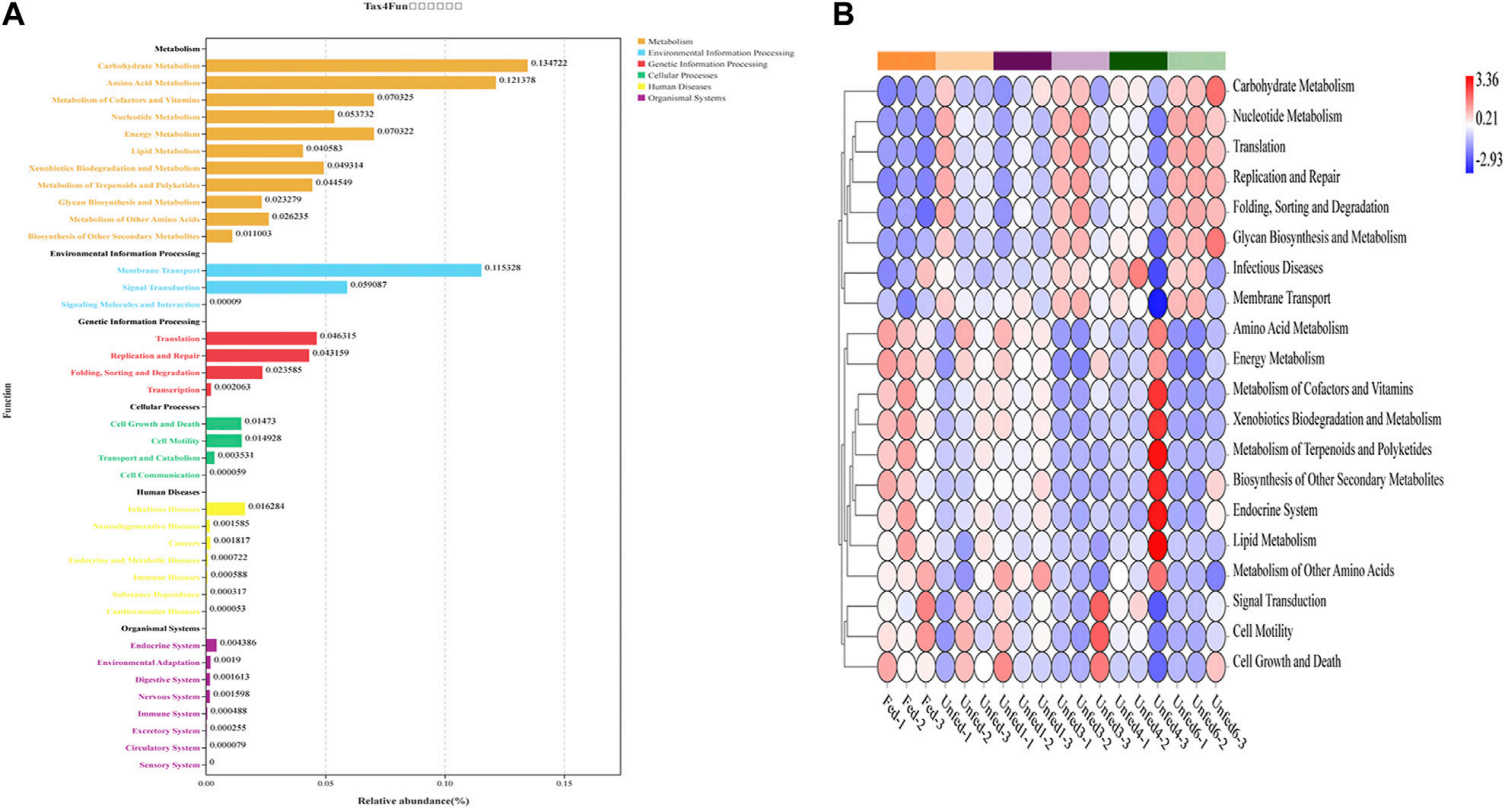
*16S rRNA* gene-predicted functional profiles obtained with Tax4Fun. **(A)**Function distribution, The vertical axis arranges the KEGG pathways of different levels, and the length of the column indicates the corresponding functional abundance in the pathway. **(B)** Heatmap, the vertical axis is functional classification, the horizontal axis is the sample, and the color indicates the abundance.

The Tax4Fun algorithm in the methods section revealed the functional profile of the gut microbiota of *Rh. sanguineus* in each group. Based on the Silva database, to better describe the changes in functional gene-related pathways in different groups, the heat map of the predicted function map of *16S rRNA* genes obtained by Tax4Fun showed the OTU annotation information of each functional level of KEGG (Figure 3). All groups showed significant changes according to KEGG functional analysis. As shown in Figure 3A, an overview of the functional distribution can be organized into six typical signaling pathways, including metabolism, environmental information processing, genetic information processing, cellular processes, human diseases, and tissue systems. As shown in Figure 3A, metabolism, environmental information processing, and genetic information processing were strongly affected in this study. The remaining three key signaling pathways were also affected to some extent.

Further analysis showed that starvation stress led to changes in all metabolic pathways. As shown in Figure 3B, starvation significantly enhanced carbohydrate metabolism, membrane transport, amino acid metabolism, cell growth, and death and cell motility compared to the fed group, suggesting that it impaired the function of translation, metabolism of cofactors and vitamins, and energy metabolism. In addition, compared with the fed group, the purine metabolism, ammonia synthesis, dimethyl ether metabolism, starch, and sucrose metabolism, amino sugar metabolism, ribosome synthesis, peptidoglycan bionucleotide sugar metabolism, and synthesis of the Unfed3, Unfed4, and Unfed6 groups signaling pathways were up-regulated. Notably, in the Unfed6 group, all other signaling pathways except arginine and proline metabolism, porphyrin and chlorophyll metabolism, oxidative phosphorylation, pyruvate metabolism, and nitrogen metabolism were up-regulated. ABC transport, purine metabolism, pyrimidine metabolism, peptidoglycan biosynthesis, arginine, proline metabolism, RNA degradation, bacterial secretion system, glycine, serine, and threonine metabolic pathways were significantly enhanced in the Unfed3 group. The acid metabolism pathway was significantly enhanced. The above results further showed that after starvation stress, the changes of microbes and signaling pathways in different parts of the gut of *Rh. sanguineus* were altered.

### 3.4 Analysis of *16S rRNA* gene tissue tropism

**FIGURE 4:**
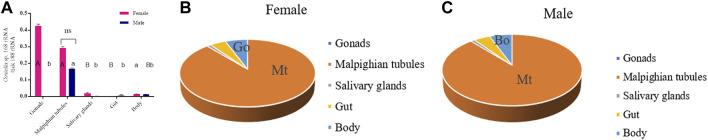
Quantification of *Coxiella* in different Rh. sanguineus organs using qPCR. **(A)** *Coxiella* in the gonads, Malpighian tubules, trunk, midgut, and salivary glands of female ticks (*n* = 15, 10, 20, 20, 19, respectively) and male ticks (*n* = 12, 10, 11, 11, 11, 11, respectively). Densities were calculated as bacterial *16S rRNA* gene targets for each tick *18S rRNA* gene target. Uppercase and lowercase letters indicate statistical significance between female and male tick organs. Red, female; blue, male; ns, not significant. **(B,C)** Relative abundance of *Coxiella* in female and male tick organs (percentage of total); orange, Malpighian tubules (Mt); pale blue, gonad.

*Coxiella* spp. density in different organs dissected from *Rh. sanguineus* ticks collected from the field were determined by qPCR. Estimated bacterial densities differed in different organs of females and males are shown in Figures 4A,B. The highest density of *Coxiella* spp. is found in female gonads. This number of bacteria was 1.4 times higher than in female Malpighian tubules, although there was no significant difference (*p* > 0.05) (Figure 4A). The densities in these two organs accounted for 97% of the total number of *Coxiella* spp. in female anatomical organs (Figure 4B). The female salivary glands, trunk (other remaining parts), and gut had a significant reduction in *Coxiella* spp. content (*p* < 0.01), averaging 1.6 orders of magnitude (Figure 4A). In male ticks, the density of *Coxiella* in the Malpighian tubules was significantly higher than in all other organs. The main difference between male and female ticks is the almost complete absence of *Coxiella* in the male gonads (Figure 4A). Although higher variation was found, on average, 97% *Coxiella* in the males were present in the Malpighian tubules (Figure 4C). Interestingly, the densities of *Coxiella* in the Malpighian tubules of male and female ticks were similar (*p* > 0.05).

### 3.5 Effects of ofloxacin injection on ticks

**FIGURE 5:**
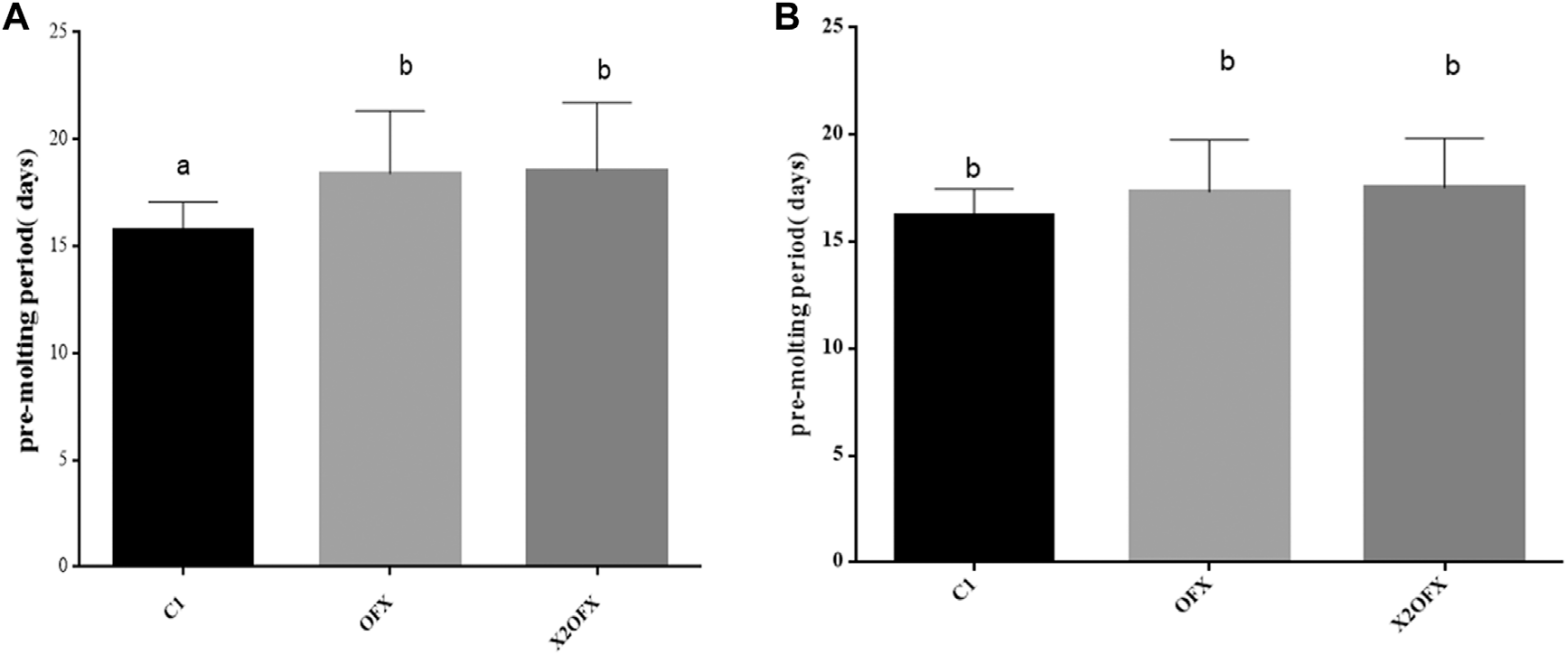
Pre-molt development time of female and male nymphs. Pre-molt development time of female **(A)** and male **(B)** nymphs, when injected with saline (C1), ofloxacin-containing saline at a dose of 30 ng/mg tick body weight (OFX) or 60 ng/mg body weight (X2OFX). For male and female nymphs, *n* = 15 per group. Different letters indicate significant differences (*p* ≤0.05).

Our experimental results showed that the molting time of female ticks was slightly prolonged after ofloxacin treatment, while the molting time of male ticks had little effect (Figures 5A,B).

**FIGURE 6:**
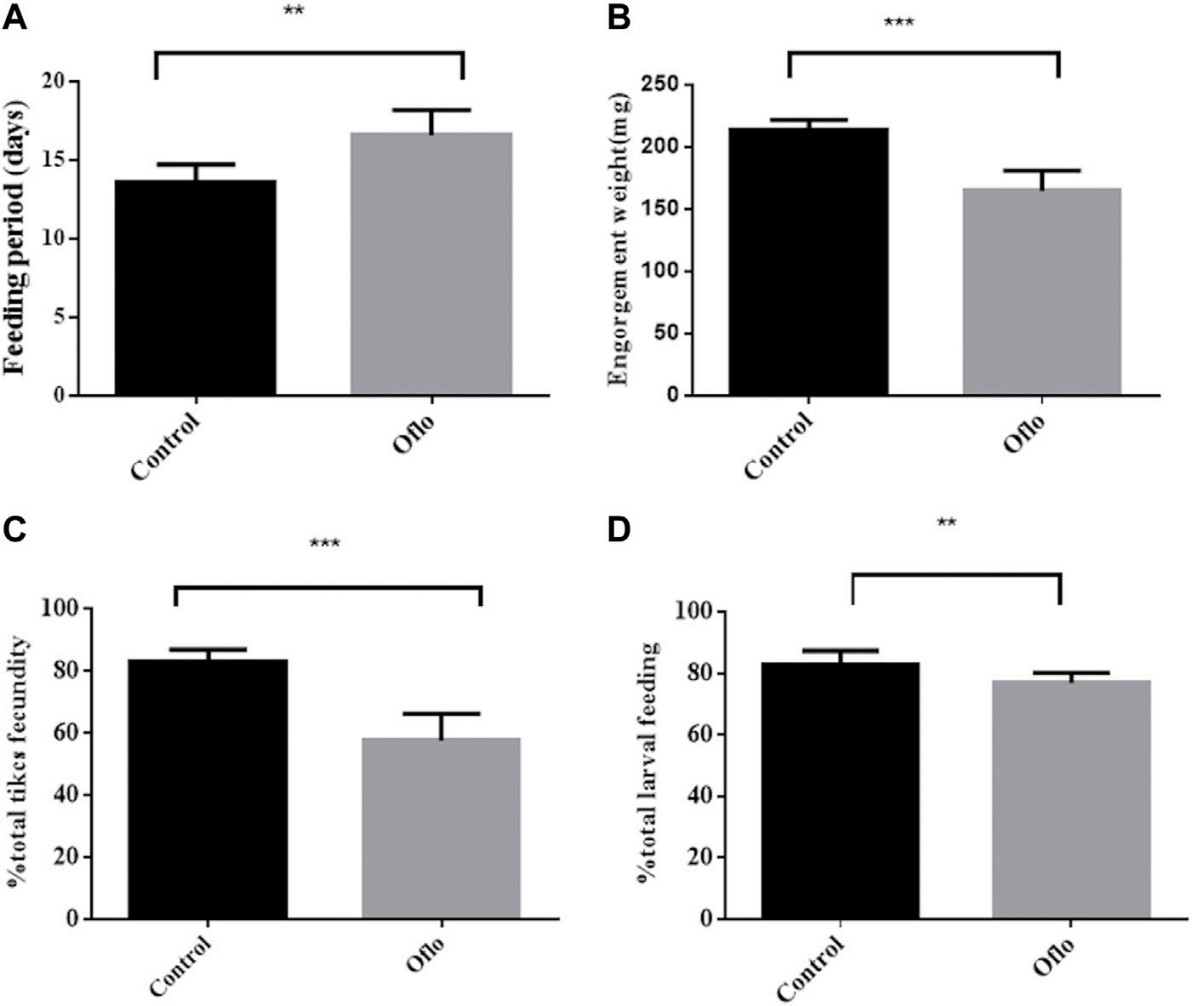
Effects of ofloxacin injection on nymphal and larval development Nymphs were injected with normal saline (control), ofloxacin-containing saline (oflo) at a dose of 30 ng/mg tick body weight (OFX). Feeding period **(A)**, engorgement weight **(B)**, fecundity **(C)**, and larval feeding rate **(D)** were shown. For male and female nymphs, *n* = 11 per group, with different letters for significant differences between groups (*p* ≤ 0.05).

On average, these female ticks finished feeding and left the host at 13.57 ± 0.43 days in New Zealand white rabbits, which was about 3 days later than the female ticks not injected with ofloxacin (the time of full blood was 16.57 ± 0.61 days), which was significantly higher in the untreated group (“Oflo”: *t* = 4.017, DF = 12, *p* < 0.0017, Figure 6A). Some ticks injected with ofloxacin could not get fully engorged, even if they stayed on the rabbit skin for a week (*n* = 7). The weight of female ticks injected with ofloxacin was approximately 76.6% of the final saturated bodyweight of the untreated group (214.2 ± 2.239 mg and 164.7 ± 4.779 mg, respectively); the weight of female ticks removed from *Coxiella* at the end of feeding was significantly lower than that of the untreated group containing saline-injected female ticks (“Oflo” *t* = 4.017, DF = 12, *p* < 0.0017, Figure 6B). Some female ticks were still alive after full blood feeding and eventually died naturally without laying eggs (*n* = 3) or shortly after leaving the host (*n* = 1). This phenomenon rarely occurs in symbiotic female ticks, suggesting that inhibition of *Coxiella* causes a significant stress on female tick feeding. All other health-related data that we quantified during the experiment were negatively affected. Compared with the normal saline group, the hatching rate of eggs in ofloxacin injected group was significantly lower (“Oflo” *t* = 8.968, DF = 20, *p* < 0.0001, Figure 6C). After the nymphs successfully molted into adults, they continued to lay eggs after full blood feeding, and calculated the changes in the larval blood-sucking rate. It was found that the blood-sucking rate of the ofloxacin injection group was 5.94% lower than that of the control group (“Oflo” *t* = 3.588, DF = 20, *p* < 0.0018, Figure 6D).

## 4 Discussion

The purpose of this study was to study the microbiome regulation of *Rh. sanguineus* under starvation stress. With the prolongation of starvation stress, the abundance of *Coxiella* gradually decreased. After using ofloxacin to eliminate *Coxiella* in the nymph, the blood-sucking rate, fecundity, and development were all affected to varying degrees. qPCR results showed that the density of *Coxiella* in the Malpighian tubules was significantly higher than those in all other organs. The main difference between male and female ticks was the almost absence of *Coxiella* in the male gonads. Although higher variability was found, on average, 97% of male *Coxiella* were present in the Malpighian tube. The density of *Coxiella* in the Malpighian tubules was similar in male and female ticks after ofloxacin injection, Pre-molt development time of female and male ticks, feeding period, engorgement weight, fecundity of the adults, and the feeding rate of the newly hatched larvae were all affected to varying degrees. Although starvation stress significantly reduced the abundance of the *Coxiella* flora, it increased relative abundance of microbial diversity in *Rh. sanguineus*.

The results of the subsequent antibiotic experiments further confirmed the effect of *Coxiella* on the development and reproduction of *Rh. sanguinis*. We did not find a dose-dependent effect of ofloxacin on nymph developmental timing, suggesting that ofloxacin has a negligible direct effect on ticks. In addition, the uniqueness of our study is the starvation stress of *Rh. sanguineus* initially demonstrated. We find that its starvation tolerance was as long as 6 months, which was not mentioned in other studies. Our findings of the microbiota pathway distribution and functional prediction of *Rh. sanguineus* under starvation stress suggest that the development and reproduction of *Rh. sanguineus* which are affected by the symbiotic *Coxiella*. Studying the distribution of symbionts and the mechanisms of nutrient metabolism may be a powerful option for controlling ticks and the pathogens that they can carry. The starved ticks had fewer endosymbionts in the midgut and salivary glands, and more in the Malpighian tubule and gonads, suggesting that either the tick is digesting the symbionts for nutrients, or the microbes are dying/moving on their own. A high density of *Coxiella*-like endosymbionts in Malpighian tubules supports a nutritional role for these symbionts, as Malpighian tubules are involved in excretion and osmoregulation. We postulate that Malpighian tubules are key organs for the nutritional symbiosis, notably the synthesis of B vitamins by *Coxiella*.

In summary, this study showed the microbial community of *Rh. sanguineus* survived for 6 months under starvation conditions in the laboratory. Therefore, the microbial community in its body changed significantly, and the symbiotic microorganism *Coxiella* regulated the development of *Rh. sanguineus* and reproduction. These findings may provide insight into implications for understanding of nutritional contribution in ticks and its consequences for tick biology and tick-borne diseases.

## Data Availability

The data presented in the study are included in the article/Supplementary Material; further inquiries can be directed to the corresponding author.

## References

[B1] Asshauer K. P. Wemheuer B. Daniel R. Meinicke P . (2015). Tax4Fun: Predicting functional profiles from metagenomic 16S rRNA data. Bioinformatics 31 (17), 2882–2884. 10.1093/bioinformatics/btv287 25957349 PMC4547618

[B2] Barker S. C. Murrell A. (2004). Systematics and evolution of ticks with a list of valid genus and species names. PARASITOLOGY 129, S15–S36. 10.1017/s0031182004005207 15938503

[B3] Ben-Yosef M. Rot A. Mahagna M. Kapri E. Behar A. Gottlieb Y. (2020). Coxiella-like endosymbiont of *Rhipicephalus sanguineus* is required for physiological processes during ontogeny. Front. Microbiol. 11, 493–509. 10.3389/fmicb.2020.00493 32390951 PMC7188774

[B4] Blisnick A. A. Foulon T. Bonnet S. I. (2017). Serine protease inhibitors in ticks: An overview of their role in tick biology and tick-borne pathogen transmission. Front. Cell. Infect. Microbiol. 7, 199–203. 10.3389/fcimb.2017.00199 28589099 PMC5438962

[B5] Caporaso J. Kuczynski J. Stombaugh J. Bittinger K. Bushman F. D. Costello E. K. (2010). QIIME allows analysis of high-throughput community sequencing data. Nat. Methods 7, 335–336. 10.1038/nmeth.f.303 20383131 PMC3156573

[B6] Chen S. Zhou Y. Chen Y. Gu J. (2018). fastp: an ultra-fast all-in-one FASTQ preprocessor. BIOINFORMATICS 34 (17), i884–i890. 10.1093/bioinformatics/bty560 30423086 PMC6129281

[B7] Dantas-Torres F. (2010). Biology and ecology of the brown dog tick. Rhipicephalus sanguineus. PARASITE VECTOR 3, 26. 10.1186/1756-3305-3-26 PMC2857863 20377860

[B8] Duron O. Morel O. Noël V. Buysse M. Binetruy F. Lancelot R. (2018). Tick-bacteria mutualism depends on B vitamin synthesis pathways. Curr. Biol. 28 (12), 1896–1902.e5. 10.1016/j.cub.2018.04.038 29861133

[B9] Edgar R. C. (2010). Muscle: Multiple sequence alignment with high accuracy and high throughput. Nucleic Acids Res. 32 (5), 1792–1797. 10.1093/nar/gkh340 PMC390337 15034147

[B10] Edgar R. C. (2013). UPARSE: Highly accurate OTU sequences from microbial amplicon reads. Nat. Methods 10 (10), 996–998. 10.1038/nmeth.2604 23955772

[B11] Gikas A. Spyridaki I. Psaroulaki A. Kofterithis D. Tselentis Y. (1998). *In vitro* susceptibility of Coxiella burnetii to trovafloxacin in comparison with susceptibilities to pefloxacin, ciprofloxacin, ofloxacin, doxycycline, and clarithromycin. Antimicrob. Agents Chemother. 42 (10), 2747–2748. 10.1128/AAC.42.10.2747 9756789 PMC105931

[B12] Guo M. Wu F. Hao G. Qi Q. Li R. Li N. (2017). *Bacillus subtilis* improves immunity and disease resistance in rabbits. Front. Immunol. 8, 354–367. 10.3389/fimmu.2017.00354 28424690 PMC5372816

[B13] Hosokawa T. Koga R. Kikuchi Y. Meng X. Y. Fukatsu T . (2010). Wolbachia as a bacteriocyte-associated nutritional mutualist. Proc. Natl. Acad. Sci. U. S. A. 107 (2), 769–774. 10.1073/pnas.0911476107 20080750 PMC2818902

[B14] Jabarit-Aldighieri N. Torres H. Raoult D. (1992). Susceptibility of *Rickettsia conorii, R. rickettsii,* and *Coxiella burnetii* to PD 127, 391, PD 131, 628, pefloxacin, ofloxacin, and ciprofloxacin. Antimicrob. Agents Chemother. 36 (11), 2529–2532. 10.1128/AAC.36.11.2529 1336950 PMC284367

[B15] Jasinskas A. Zhong J. Barbour A. G. (2007). Highly prevalent *Coxiella* sp. bacterium in the tick vector *Amblyomma americanum* . Appl. Environ. Microbiol. 73, 334–336. 10.1128/AEM.02009-06 17085709 PMC1797106

[B16] Lalzar I. Harrus S. Mumcuoglu K. Y. Gottlieb Y. (2012). Composition and seasonal variation of *Rhipicephalus turanicus* and *Rhipicephalus sanguineus* bacterial communities. Appl. Environ. Microbiol. 78, 4110–4116. 10.1128/AEM.00323-12 22467507 PMC3370557

[B17] Latrofa M. S. Dantas-Torres F. Giannelli A. Otranto D. (2014). Molecular detection of tick-borne pathogens in *Rhipicephalus sanguineus* group ticks. Ticks Tick. Borne. Dis. 5, 943–946. 10.1016/j.ttbdis.2014.07.014 25113982

[B18] Lockwood S. Brayton K. A. Broschat S. L. (2016). Comparative genomics reveals multiple pathways to mutualism for tick-borne pathogens. BMC GENOMICS 17, 481. 10.1186/s12864-016-2744-9 27368698 PMC4930560

[B19] Nikoh N. Hosokawa T. Moriyama M. Oshima K. Hattori M. Fukatsu T. (2014). Evolutionary origin of insect–Wolbachia nutritional mutualism. Proc. Natl. Acad. Sci. U. S. A. 111 (28), 10257–10262. 10.1073/pnas.1409284111 24982177 PMC4104916

[B20] Ninio C. Plantard O. Serra V. Pollera C. Ferrari N. Cafiso A. (2015). Antibiotic treatment of the hard tick *Ixodes ricinus*: Influence on *Midichloria mitochondrii* load following blood meal. Ticks Tick. Borne. Dis. 6, 653–657. 10.1016/j.ttbdis.2015.05.011 26055234

[B21] Ondov B. D. Bergman N. H. Phillippy A. M. (2011). Interactive metagenomic visualization in a Web browser. BMC Bioinforma. 12, 385. 10.1186/1471-2105-12-385 PMC3190407 21961884

[B22] Price M. N. Dehal P. S. Arkin A. P. (2010). FastTree 2--approximately maximum-likelihood trees for large alignments. PLOS ONE 5 (3), e9490. 10.1371/journal.pone.0009490 20224823 PMC2835736

[B23] Salzberg S. L. (2011). Flash: Fast length adjustment of short reads to improve genome assemblies. BIOINFORMATICS 27 (21), 2957–2963. 10.1093/bioinformatics/btr507 21903629 PMC3198573

[B24] Wickham H. Boussiala M. (2020). R packages organize, test, document, and share your code. Sebastopol, CA: O’Reilly books O’Reilly Media.

[B25] Zhang C. M. Li N. X. Zhang T. T. Qiu Z. X. Li Y. Li L. W. (2017). Endosymbiont CLS-HI plays a role in reproduction and development of *Haemaphysalis longicornis* . Exp. Appl. Acarol. 73, 429–438. 10.1007/s10493-017-0194-y 29197022

[B26] Zhong Z. Zhong T. Peng Y. Zhou X. Wang Z. Tang H. (2021). Symbiont-regulated serotonin biosynthesis modulates tick feeding activity. Cell Host Microbe 29, 1545–1557.e4. 10.1016/j.chom.2021.08.011 34525331

