# Supplementary material for: The microbiota changes of the brown dog tick, Rhipicephalus sanguineus under starvation stress
Source: Front Physiol. 2022 Sep 9;13:932130. doi: 10.3389/fphys.2022.932130 (PMC9504665; doi:10.3389/fphys.2022.932130)
Supplement: Supplementary file 1 [file Table1.DOCX]

**Supplementary information**

**Table S1 PCR primers for bacterial 16S rRNA genes used in this stud**y

| Sequencing region | primer name | Sequence |
| --- | --- | --- |
| 341F-806R | 341F | CCTACGGGNGGCWGCAG |
|  | 806R | GGACTACHVGGGTATCTAAT |

**Table S2   PCR primers for qPCR used in this study**

| Primer | Sequence | Gene | Target organisms |
| --- | --- | --- | --- |
| Cox sp434f | 5’- CCTTTTGAGCGTTGACGTTA-3’ | 16S rRNA | *Coxiella* |
| Cox sp1004r | 5’- CCAAAGGCACCAAGTCATTT -3’ | 16S rRNA | *Coxiella* |
| SNR | 5'-AATTGACATCCTATTTCAAA-3' | 12S rRNA | *Rh. sanguineus* |
| T2AR | 5’-AATGAGAGCGACGGGCGATGT-3’ | 12S rRNA | Arthropoda |

**Table S3 Data preprocessing statistics and quality control**

| Sample Name | Raw PE | Clean PE | Raw Tags | Clean Tags | Chimera | Effective Tags | Effective Ratio (%) |
| --- | --- | --- | --- | --- | --- | --- | --- |
| Fed-1 | 130710 | 129999 | 112086 | 110403 | 84 | 110319 | 84.40 |
| Fed-2 | 132939 | 132284 | 112858 | 111102 | 68 | 111034 | 83.52 |
| Fed-3 | 122944 | 122318 | 106010 | 104307 | 91 | 104216 | 84.77 |
| Unfed-1 | 129435 | 128687 | 113823 | 111648 | 198 | 111450 | 86.10 |
| Unfed-2 | 132727 | 132065 | 115115 | 113468 | 98 | 113370 | 85.42 |
| Unfed-3 | 120024 | 119393 | 103669 | 102128 | 71 | 102057 | 85.03 |
| Unfed1-1 | 137175 | 136206 | 121026 | 117810 | 1587 | 116223 | 84.73 |
| Unfed1-2 | 132625 | 131705 | 116787 | 113598 | 147 | 113451 | 85.54 |
| Unfed1-3 | 135330 | 134496 | 117510 | 114884 | 124 | 114760 | 84.80 |
| Unfed3-1 | 126492 | 125833 | 109676 | 108476 | 270 | 108206 | 85.54 |
| Unfed3-2 | 133616 | 132607 | 116693 | 113934 | 241 | 113693 | 85.09 |
| Unfed3-3 | 130969 | 130126 | 117242 | 112720 | 2319 | 110401 | 84.30 |
| Unfed4-1 | 128026 | 127119 | 111992 | 108996 | 125 | 108871 | 85.04 |
| Unfed4-2 | 120735 | 119759 | 106149 | 103140 | 169 | 102971 | 85.29 |
| Unfed4-3 | 125067 | 124392 | 111065 | 102867 | 124 | 102743 | 82.15 |
| Unfed6-1 | 121660 | 120826 | 106329 | 104174 | 85 | 104089 | 85.56 |
| Unfed6-2 | 121536 | 120638 | 106696 | 104438 | 105 | 104333 | 85.85 |
| Unfed6-3 | 133397 | 132744 | 113392 | 111472 | 159 | 111313 | 83.44 |
